# Measuring Vulnerability for Small Areas

**DOI:** 10.23889/ijpds.v9i5.2918

**Published:** 2024-09-18

**Authors:** Susan McVie, Ana Morales-Gomez

**Affiliations:** 1 University of Edinburgh; 2 Scottish Centre for Administrative data Research (SCDAR); 3 University of Sheffield

## Objective

In Scotland, the link between alcohol consumption and violent crime remains a significant concern. This study aims to explore the association between assault-related incidents captured by health services in Scotland and the availability and density of alcohol premises in Scotland.

## Approach

Scotland has experienced an overall decline in violence in the last years; however, this trend varies significantly across different regions. Research has shown the importance of analysing the spatial and social contexts in which violence occurs. Using linked administrative data from ambulance services and a geographically aggregated dataset of the location of licensed outlets selling alcohol, we explore the link between place, alcohol premises densities and violence using regression models.

## Results

Our findings show that violent incidents are concentrated in the most deprived areas and places with higher availability of alcohol premises. However, areas with a higher number of off-sales premises showed a higher prevalence of violent incidents than those with a higher number of on-sales premises. In addition, violent incidents exhibit an uptick during weekends, evenings, and early mornings, suggesting a link to the night-time economy.

## Conclusions and Implications

The persistence of violence in Scotland, particularly in deprived areas with high concentrations of alcohol outlets, emphasise the need for public policies and interventions aimed at mitigating the adverse effects of alcohol-related violence. Data linkage between health services and police can contribute to understanding the spatial and social contexts in which violence occurs and could inform the design of focused crime prevention and public health approaches.

